# Psychometric Properties of the CASP-12 Scale in Portugal: An Analysis Using SHARE Data

**DOI:** 10.3390/ijerph17186610

**Published:** 2020-09-11

**Authors:** Carmen Rodríguez-Blázquez, Oscar Ribeiro, Alba Ayala, Laetitia Teixeira, Lia Araújo, Maria João Forjaz

**Affiliations:** 1 National Centre of Epidemiology, Carlos III Health Institute, 28029 Madrid, Spain; crodb@isciii.es (C.R.-B.); jforjaz@isciii.es (M.J.F.); 2 Center for Health Technology and Services Research (CINTESIS), Department of Education and Psychology of the University of Aveiro, 3810-193 Aveiro, Portugal; 3 National School of Public Health, Carlos III Health Institute, 28029 Madrid, Spain; arwen.alba@gmail.com; 4 Center for Health Technology and Services Research (CINTESIS), Institute of Biomedical Sciences Abel Salazar, University of Porto (ICBAS.UP), 4050-313 Porto, Portugal; laetitiateixeir@gmail.com; 5 Center for Health Technology and Services Research (CINTESIS), School of Education, Polytechnic Institute of Viseu (ESEV.IPV), 3504-510 Viseu, Portugal; liajaraujo@esev.ipv.pt

**Keywords:** quality of life, CASP-12, Rasch model, older adults, Portugal

## Abstract

The purpose of this study is to assess the psychometric properties of the Portuguese version of the Control, Autonomy, Self-realization, and Pleasure (CASP)-12 scale used in the Survey of Health, Aging and Retirement in Europe (SHARE) project. Data were obtained from a representative sample of 1666 people aged ≥50 years living in Portugal and participating in the SHARE wave 6. In addition to the CASP-12 scale, sociodemographic data and health status, activity limitation (GALI), depression (Euro-D) and satisfaction with life scores were collected. Data quality and acceptability, construct and structural validity and internal consistency of the CASP-12 scale were analyzed. A Rasch analysis was also performed. CASP-12 total score (mean: 33.3; standard deviation: 5.8, range: 12–48) correlated with Euro-D (−0.57) and with life satisfaction (0.52). Mean scores were significantly lower for women, people aged ≥75 years and those with activity limitations and worse health status (*p* < 0.001). The confirmatory factor analysis showed good fit to the 4-factor model (root mean squared error of approximation (RMSEA): 0.07; comparative fit index (CFI): 0.90, χ^2^ (48) = 444.59, *p* < 0.001), which was confirmed by Rasch analysis (χ^2^ (36) = 10.089, *p* = 0.745, person separation index (PSI) = 0.722 for the 4-factor model). For domains, person separation index ranged 0.31–0.79 and Cronbach’s alpha, 0.37–0.73. In conclusion, the Portuguese version of the CASP-12 scale presents some inadequacies in acceptability, internal consistency and structural validity.

## 1. Introduction

By 2050, Portugal is projected to be one of the oldest countries in Europe, with persons aged 55 years or more representing almost half (47.1%) of the total population [1]. This increasing population aging raises many questions at socioeconomic, health, scientific and ethics levels. It is fundamental to consider in which conditions and with what quality of life (QoL) older adults are and will be living. This important question has led to international interest in the enhancement, and measurement, of quality of life in old age, attracting increasing research and policy interest [2]. Specifically, in Portugal, the advancement in aging research is noteworthy, with some studies approaching conceptualization and assessment of QoL and related concepts, such as well-being, successful and active aging.

The World Health Organization Quality of Life (WHOQOL) group has developed outstanding work in this area. This group has proposed a definition and operationalized the construct from a cross-cultural perspective, resulting in three instruments: WHOQOL-100, WHOQOL-BREF and WHOQOL-OLD [3,4]. This last one was specifically created to study older adults’ QoL and already has a European Portuguese version, with the same categories of the original version (sensory abilities; autonomy; past, present and future activities; social participation; death and dying; and intimacy) and with a seventh one regarding family/family life, which is an important dimension for understanding Portuguese older adults’ QoL [5]. Despite the common use of WHOQOL instruments in research and the availability of several measures to assess health-related QoL in Portugal (e.g., SF-36, EQ-5D), the extent of available generic QoL instruments applicable to the older population is still limited.

The Control, Autonomy, Self-realization, and Pleasure (CASP)-19 scale is one of most frequently used measures of QoL in older age [6]. Based on a psycho-sociological conceptualization of QoL that draws on the “theory of human need”, scale includes 19 Likert-type items that reflect four different dimensions of QoL: control (four items), autonomy (five items), self-realization (five items), and pleasure (five items) [6,7,8]. Taken together, the items can be combined into a measure of overall QoL, as they present the degree to which the respondents have their needs covered. Due to the questionable psychometric properties of CASP-19 presented by several researchers who highlighted that the factor structure of the scale did not follow what was predicted by the theory [9,10], a shorter version of the scale has been proposed (CASP-12) [9]. This version leaves out the items that only weakly reflect a given dimension [9].

To date, both the original version and the shorter version of CASP have been increasingly used internationally. The scale has been included in many national and cross-national studies in countries from North and South America, as well as from Africa and Australasia [11]. In Europe, it has been used in countries like United Kingdom, France, Sweden and Ireland, being also included in two well-known cross-national projects: the Health, Alcohol and Psychosocial factors in Eastern Europe (HAPIEE) and the Survey of Health, Aging and Retirement in Europe (SHARE) [12]. This last project in particular constitutes a longitudinal survey conducted every two years since 2004 that collects information on medical, social and economic status of the population aged 50+ from more than 20 countries in Europe [13].

In a recent and thorough examination of the psychometric properties of the SHARE version of the CASP-12 scale that included internal consistency analyses, factor structure analyses, item-total Spearman correlations, and cross country invariance, Borrat-Besson et al. showed that the structure postulated by the authors of the CASP-12 scale could not be replicated, and suggested a revised scale with ten items instead of twelve [14]. In addition, when exploring the cross-cultural robustness of the results, they showed that Portugal and Italy presented different results. Both countries stand out against the general results pattern with respect to the pleasure dimension since item 10 (“I look forward to each day”) correlated negatively with all dimensions. This result was associated to aspects of translation and interpretation, reinforcing the importance of cultural significance in validation processes. Other studies have found a 2 or 3-factor structure [15,16].

Presently, the number of studies advancing the available understanding of the underlying structure of the CASP scale has been rising [11], including the evaluation of its dimensionality using Rasch analysis [17]. Rasch analysis is a robust item response theory technique that provides information on the dimensionality of a scale, contributing thus to the construct validity testing, and also on item fit and item local dependency, differential item functioning (DIF), and disordered response categories. Scales that fit the Rasch model provide an interval linear measure that establishes equal intervals between the values, and that could be used in parametric analysis. Despite these advances, there are no full validation studies of the Portuguese version of the CASP scale used in the SHARE project.

The purpose of this study is to analyze the psychometric properties of the European Portuguese version of the CASP-12 scale used in the SHARE project in a sample of people aged 50 years of older.

## 2. Materials and Methods

### 2.1. Study Design and Setting

A cross-sectional, national study in a sample of people aged 50 years or older living in Portugal and participating in the wave 6 (W6, 2015) SHARE project.

### 2.2. Participants

The sample was composed by 1666 people aged 50 or older at the time of sampling, living in Portugal and recruited for the W6 of the SHARE project [18]. Participants were excluded if they were incarcerated, hospitalized or out of the country during the entire survey period, unable to speak the country’s language or had moved to an unknown address. Detailed information on the sample and data collection can be found in Malter and Börsch-Supan [19].

The SHARE project was reviewed and approved by the Ethics Council of the Max Planck Society. The Ethics Committee of Carlos III Institute of Health approved the present study (reference: CEI PI 62-2019).

### 2.3. Measures

The main outcome variable was the QoL measured with the CASP-12 scale [9,13], a 12-item self-assessed questionnaire. Four domains compose the scale: control, autonomy, self-realization, and pleasure. Items are rated on a four-point Likert scale from 1 = never to 4 = often, although item 4 and items 7 to 12 are reversed, thus, the lowest scores mean the worst QoL. The total score ranges from 12 to 48, with higher scores meaning better QoL.

For depression, the Euro-D was applied [20]. It is a self-completed scale with items assessing depression, pessimism, suicidality, guilt, sleep, interest, irritability, appetite, fatigue, concentration (on reading or entertainment), enjoyment, and tearfulness. The Self-perceived Health—United States version (SPHUS) [21] was applied for health status (rated from 1, excellent, to 5, poor). The Global Activity Limitation Indicator (GALI) [22], which is a single-item (“For the past 6 months at least, to what extent have you been limited because of a health problem in activities people usually do?”) with limited and non-limited categories for self-perceived activity limitation. An item on life satisfaction (“On a scale from 0 to 10 where 0 means completely dissatisfied and 10 means completely satisfied, how satisfied are you with your life?”) and a checklist of chronic diseases were also used.

Socio-demographic variables (age, sex, marital status, job situation, years of education and living in urban or rural setting) were also compiled and used to characterize the sample. Education level was described according to the International Standard Classification of Education (ISCED) [23]. A description of all variables used in SHARE W6 can be found in Börsch-Supan [18]. Items included in the SHARE survey were translated into European Portuguese following a common procedure [24].

### 2.4. Statistical Analysis

The outcome variable—CASP-12 total score—did not fit normal distribution (Kolmogorov–Smirnov test with Lilliefors correction, *p* < 0.001), and non-parametric statistics were applied. Descriptive statistics were used for characterizing the sample in terms of socio-demographic variables and rating scales scores.

The psychometric properties of CASP-12 were explored using classical test theory (CTT) and Rasch analysis, a variation of item response theory (IRT). According to the CTT principles, the following psychometric attributes were calculated: data quality and acceptability, construct (structural and hypotheses testing) validity, and reliability (internal consistency) [25,26].

For data quality and acceptability, the percentage of missing data (standard criterion: ≤15%) for CASP-12 items, mean, median, standard deviation (SD), range of observed versus theoretical values, skewness (criterion: −1 to +1), floor and ceiling effects (criterion: ≤15%) of the CASP-12 items and total score were calculated [27].

For structural validity, a confirmatory factorial analysis (CFA) using maximum likelihood estimations was performed. For CFA, a root mean squared error of approximation (RMSEA) ≤ 0.06 and a comparative fit index (CFI) > 0.9 indicated good fit to the model [28]. Additionally, for structural validity, the corrected item-total correlation (criterion: ≥0.20) and the inter-correlation of CASP-12 domains (internal validity) using Spearman’s rank correlation coefficients were calculated.

Testing of hypotheses comprises convergent and discriminative validity. Convergent validity was calculated using the Spearman’s rank correlation coefficients of CASP-12 total and domain scores with the remaining measures. Based on previous studies, a moderate correlation was hypothesized between CASP-12 and depression (Euro-D), activity limitation (GALI) and life satisfaction (r_S_ = 0.30–0.50) [29]. Discriminative or known-groups validity was explored by calculating the differences in CASP-12 scores in the sample grouped by variables of interest: sex, age group (50 to 64, 65 to 74, and 75 years and older), and with or without activity limitation (GALI). Mann–Whitney or Kruskal–Wallis tests were used to ascertain differences between groups.

Internal consistency was explored by computing Cronbach’s alpha (criterion: ≥0.70) and the item homogeneity index (criterion: >0.30) [30].

For Rasch analysis, the following attributes were calculated: fit to the Rasch model [31], unidimensionality, internal consistency (person separation index, PSI), item local independency, absence of differential item functioning (DIF) by age (3 groups) and sex, and threshold ordering. There are excellent publications where detailed information is provided to the lay reader about how to conduct a Rasch analysis and interpret its results [32,33,34].

Fit to the Rasch model was considered when there was a non-significant chi-square value with Bonferroni adjustment for a number of items. Furthermore, item and person fit residuals should follow a distribution with mean of 0 and standard deviation of 1, with individual item fit residuals being expected to fall within the −2.5 to 2.5 range. For unidimensionality, the person estimates of two sets of items defined in a principal component analysis of the residuals are compared through t-tests. For a scale to be unidimensional, the lower bound of the binomial confidence interval should overlap 5% [35,36].

Reliability was measured with the PSI, which is interpreted similarly to Cronbach’s alpha. Item local independency is ascertained when the item corrections are low (<0.30 of the mean correlations) following removal of the variance due to the first Rasch factor. Locally dependent items may be combined into a single, super item [37].

DIF was measured with an analysis of variance [38]. In the case of uniform DIF, the item may be split, and item locations are calculated separately by each group. Thresholds are points between two response categories with equal probability of answer. Threshold ordering means that the participants use the response categories in an expected way, consistent with the construct continuum. In the case of threshold disordering, two adjacent response categories are collapsed.

We followed an iterative process, where model modifications were made and repeatedly tested until model specifications were met [33,39]. Large sample sizes provide a high statistical power that will determine small deviations from the Rasch model as statistically significant. Therefore, a random sample of 300 cases was taken and analyzed [40].

Statistical significance was set at *p* < 0.05. CTT calculations were performed using IBM SPSS Statistics 22.0 (IBM, Armonk, NY, USA), except CFA, for which Stata 14.0 was used. Rasch analyses were performed using RUMM2030 [41].

## 3. Results

The sample was composed of 55% women, had an overall mean age of 67.81 (SD: 9.01; range: 50–94), and presented an average of 6.28 years of education (SD: 4.16; range: 0–25), with most of the sample (61.8%) having primary level education. Most of the participants were married or lived with a spouse (75.9%), were retired (62.4%), and lived in urban settings (72.5%). A description of the sample is presented in Table 1.

The CASP-12 total score was computable for 1468 (88.1%) participants, thus, missing data represented 11.9% of the sample. The mean CASP-12 was 26.68 (SD: 5.80, range: 12–48). Skewness for the total score was 0.311, and floor and ceiling effects were less than 0.5% (Table 2). For domains, pleasure presented the highest percentage of missing data (11.3%) and floor effect (12.1%). All domain scores covered the full score range (3 to 12 points). No domain showed a ceiling effect. Some items presented marked floor or ceiling effects, particularly in the autonomy and pleasure domains.

**Figure 1:**
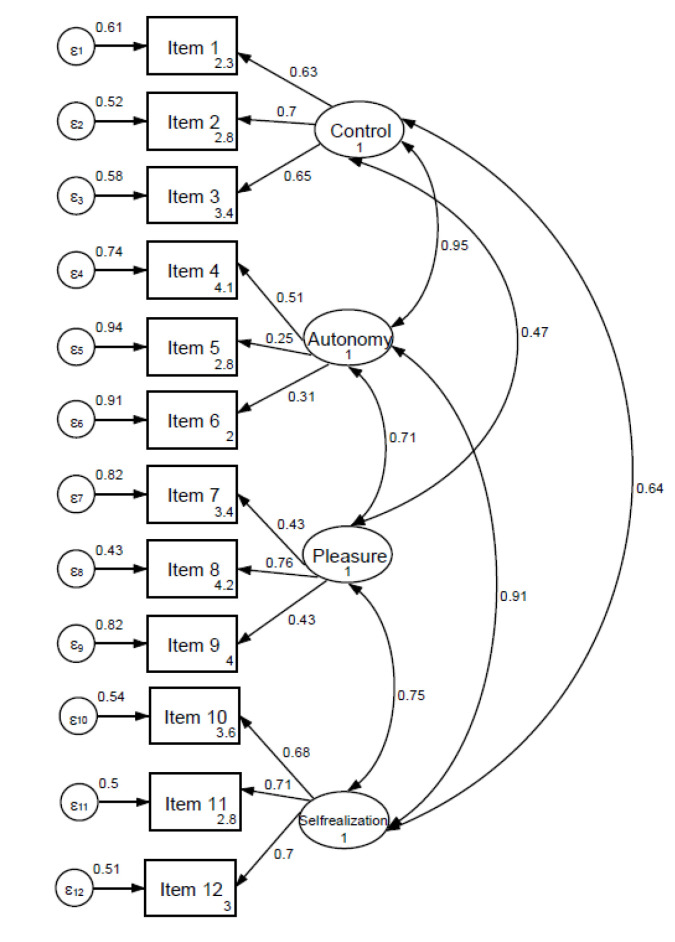
Path diagram of the CASP-12 model. Root mean squared error of approximation (RMSEA) = 0.07; comparative fit index (CFI) = 0.90 (χ^2^ = 444.59; df = 48; *p* < 0.001). The diagram shows standardized values of loadings and error variance.

Figure 1 shows the path diagram of the CASP-12 scale performed through CFA. The 4-factor model obtained a RMSEA of 0.07 and a CFI of 0.90, χ^2^ (48) = 444.59, *p* < 0.001). Details are provided in the Supplementary Material, including models for one and three factors.

Item–total corrected correlation was 0.25–0.59 for total scale. By domains, the lowest values corresponded to autonomy (0.13–0.26) and the highest to self-realization (0.48–0.61). The inter-domain correlation coefficients (internal validity) are shown in Table 3. CASP-12 domains correlated from 0.22 (autonomy and pleasure) to 0.46 (pleasure and self-realization). Regarding convergent validity (Table 3), CASP-12 total score correlated −0.57 with Euro-D, 0.52 with life satisfaction, −0.47 with self-perceived health and 0.41 with GALI. For domains, control (r_S_ = 0.49) and self-realization (r_S_ = 0.46) reached the highest correlation coefficients with Euro-D. Self-realization also showed the highest correlation coefficients with life satisfaction (r_S_ = −0.46) and self-perceived health (r_S_ = 0.45). The pleasure domain displayed the lowest correlation coefficients with the other applied measures.

CASP-12 total and domains showed significant differences in scores by sex (women showed lower mean scores than men, *p* < 0.01), by age groups (lower mean scores in older participants in all domains except in autonomy, *p* < 0.01), by activity limitation assessed with GALI (higher mean scores for those participants without limitations, *p* < 0.001), by self-perceived health (lower mean scores for participants with poor health, *p* < 0.001), and by presence of multimorbidity (lower mean scores for participants with two or more chronic conditions, *p* < 0.001) (Table 4).

Regarding internal consistency (Table 5), Cronbach’s alpha for the total score of CASP-12 was 0.78, with a range from 0.37 (autonomy) to 0.73 (self-realization) for domains. Inter-item correlation and item homogeneity indexes were lower for autonomy and higher for self-realization.

Finally, a Rasch analysis was performed with the 12 items, showing a lack of fit to the Rasch model. Therefore, each domain was analyzed separately, and all showed unidimensionality, ordered thresholds, item local independency and lack of DIF by gender.

Table 6 presents the person and item fit parameters for the CASP-12 domains. The control domain presented a good fit to the Rasch model, PSI = 0.617. Item 1 (“My age prevents me from doing the things I would like to do”) displayed DIF by age, with adults aged 75 or more underestimating scores. Similarly to control, the autonomy domain, showed a good fit to the Rasch model with a low PSI of 0.312 and DIF by age for item 4 (“I can do the things I want to do”). The pleasure domain displayed an adequate fit to the Rasch model after splitting items 8 (“I feel that my life has meaning”) and 9 (“On balance, I look back on my life with a sense of happiness”) due to DIF by age (underestimation of scores by older adults), with low PSI (0.372). Finally, the self-realization section had a good fit to the Rasch model, PSI = 0.71, and no DIF by age groups. When super items were created for each domain, a good fit to the Rasch model was observed, with χ^2^ (36) = 10.089, *p* = 0.745, PSI = 0.722.

## 4. Discussion

This is the first complete validation study with the European Portuguese version of the CASP-12 scale used in the SHARE study. This version is slightly different to that originally proposed by Wiggins et al. [9].

Regarding data quality and acceptability, missing data and skewness of domains and total score were within the standard limits. Most items showed a marked ceiling effect, particularly in the control and pleasure domains, and two items showed a floor effect. This is in line with the other studies reporting the distribution of items scores in CASP-12 [15]. However, domains and total score did not show floor or ceiling effects.

Internal consistency of CASP-12 was satisfactory in the self-realization domain and when combining all items. The autonomy domain presented the lowest values of internal consistency, as reported in previous studies [14,15]. The autonomy and pleasure domains also showed low reliability indices in Rasch analysis. This should be considered when using these two domains individually.

The autonomy domain also showed the weakest results for internal, construct and structural validity. This domain showed low correlation coefficients with other domains (0.22 with pleasure and 0.36 with self-realization). In Rasch analysis, the autonomy domain presented a low reliability and one item with bias by age. Some authors have proposed dropping the items on “family responsibilities” and on “shortage of money”, as they could be measuring something different to QoL [9], or forming a domain with control in a bi- or tri-factorial model [15,16]. However, despite the problems with the item on “family responsibilities”, family life is an important dimension for Portuguese older adults’ QoL, as commented in the Introduction, and this prevent us from deleting this item [5].

Moreover, the CFA supported the 4-factor model proposed by CASP-12 original developers [42]. However, the factor intercorrelations found in the CFA were much higher than the scale intercorrelations for internal validity. In addition, items 5 and 6 items loaded weakly on the “autonomy” scale, and the same was observed for item 7 on the “pleasure” scale. This suggests the need for further research to elucidate on the advantages and disadvantages of using different factor structures of the CASP-12 scale in Portugal. Even though the first 12-item model did provide a good fit to the Rasch model, an adequate fit was found for each of the individual domains, as well as the model with one super item per domain. Thus, results from Rasch analysis point to a hierarchical scale structure of the CASP-12 scale with a higher-order construct, QoL, formed by four lower-order unidimensional sections. A previous Rasch analysis found evidence for a unidimensional 15-item version of the CASP scale [17].

Regarding convergent validity, the total score of CASP-12 showed the highest correlation coefficients with the item on satisfaction with life and with the Euro-D, as hypothesized. Depression is a main determinant of QoL deterioration, and this relationship has been previously reported in other studies applying CASP-12 in older samples [43].

Women, participants aged 76 year or older, with limitations of activity, with poor self-perceived health and with two or more chronic diseases scored significantly lower in CASP-12, as in other studies [43,44]. Portugal has a high old age–dependency ratio, reaching 65.8% [1]; thus, these results suggest the need of intervention in the most vulnerable population to improve their QoL and achieve healthy and active aging. Gender differences are not due to an item bias by gender, as indicated in our DIF analyses and in previous studies [17]. However, a bias by age was found in four items, and further research is needed to confirm our findings. If confirmed, differences by age in the autonomy and pleasure dimensions should be interpreted cautiously, as they might be due to item bias.

Several limitations to this study must be acknowledged. A heterogeneous, diverse sample is usually advised for validation studies. In this case, the SHARE project does not include individuals living in nursing homes. Furthermore, because of the cross-sectional design, it is not possible to evaluate the temporal stability of the structure presented. Therefore, further studies should assess CASP-12 validity with other Portuguese samples (i.e., institutionalized older adults) and analyze responses for test-retest reliability.

In explicitly resisting a conflation of QoL with health status that often happens in old age, the CASP scale was developed to focus on favorable and advantageous features of aging and on older people’s positive characteristics [42]. Despite some limitations, it is an instrument with adequate psychometric features, and its use is encouraged as it may contribute to furthering the study of older adults’ needs and strengths and therefore improve the well-being of the older population in Portugal. The CASP-12 scale is a QoL instrument that might be useful for clinical practice, as well as to assess public health interventions and aging policies in Portugal. In addition, this study underscores the past and future research studies performed with data from the SHARE project and that use the CASP-12 scale as a QoL measure.

## 5. Conclusions

In conclusion, the European Portuguese version of the CASP-12 scale, when applied to people aged 50 years or older, presented some inadequacies in terms of acceptability, internal consistency and structural and construct validity of two of the four domains that compose it. Therefore, the total score could be preferred over the use of individual domains scores. The European Portuguese version of the CASP-12 scale used in the SHARE project presents some strengths, such as good acceptability, unbiased scores by gender, fit to the Rasch model, and adequate reliability of the pleasure and self-realization domains. Nevertheless, future research should present more evidence on the scale’s psychometric properties, including its factor structure in different samples, namely, old–old and institutionalized individuals.

## Figures and Tables

**Table 1:** Characteristics of the sample.

Variable	Categories	*n*	%
Gender	Male	749	45.0
	Female	917	55.0
Age groups	50–64 years old	653	39.2
	65–74 years old	643	38.6
	>75 years old	360	22.2
Marital status	Married—living with spouse	1264	75.9
	Registered partnership	37	2.2
	Married—not living with spouse	22	1.3
	Never married	53	3.2
	Divorced	72	4.3
	Widowed	218	13.1
Education	None	101	6.0
	Primary education	1036	61.8
	Lower secondary education	171	10.2
	Upper secondary education	156	9.3
	University	162	9.7
	Other	49	2.9
Job situation	Retired	1021	62.4
	Employed	256	15.6
	Homemaker	131	8.0
	Other	229	14.0
Setting	Urban	687	72.5
	Rural	260	27.5
Self-perceived health	Excellent, very good or good	527	31.6
	Fair	781	46.9
	Poor	358	21.5
GALI	Not limited	671	40.3
	Limited	995	59.7
Number of chronic diseases	0 or 1	596	35.8
	2 or more	1070	64.2
**Variable**	**Observed minimum–maximum**	**Mean**	**SD**
Age	50–94	67.81	9.01
Years of education	0–25	6.28	4.16
Euro-D	0–12	3.46	2.67
Life satisfaction	0–10	7.05	2.24

GALI: Global Activity Limitation Indicator; SD: standard deviation.

**Table 2:** Data quality and acceptability of CASP-12.

How Often Do You Feel/Think	Missing %	Mean	Median	SD	Skewness	Min	Max	Floor Effect %	Ceiling Effect %
1. Your age prevents you from doing the things you would like to do?	10.7	2.63	3.00	1.03		1	4	19.4	22.2
2. That what happens to you is out of your control?	10.9	2.46	2.00	0.91		1	4	15.7	12.9
3. Left out of things?	10.7	1.91	2.00	0.91		1	4	40.6	6.1
CASP-12 Control	11.2	7.00	7.00	2.24	0.19	3	12	7.5	3.2
4. That you can do the things that you want to do? (R)	10.5	1.79	2.00	0.79		1	4	40.9	3.4
5. That family responsibilities prevent you from doing what you want to do?	10.6	2.23	2.00	1.01		1	4	29.4	12.4
6. That shortage of money stops you from doing the things you want to do?	10.5	3.09	3.00	0.97		1	4	8.9	43.3
CASP-12 Autonomy	10.6	7.10	7.00	1.85	−0.06	3	12	3.4	0.7
7. Look forward to each day? (R)	11.2	2.11	2.00	0.85		1	4	25.5	5.9
8. That your life has meaning? (R)	10.7	1.68	1.00	0.80		1	4	50.3	2.9
9. Look back on your life with a sense of happiness? (R)	10.6	1.80	2.00	0.81		1	4	40.6	3.9
CASP-12 Pleasure	11.3	5.59	5.00	1.77	0.63	3	12	12.1	0.5
10. Full of energy these days? (R)	10.5	2.03	2.00	0.83		1	4	27.4	7.2
11. That life is full of opportunities? (R)	10.8	2.53	3.00	0.87		1	4	12.2	13.5
12. That the future looks good for you? (R)	11.0	2.45	2.00	0.85		1	4	12.1	11.3
CASP-12 Self-realization	11.2	7.01	7.00	2.05	0.17	3	12	5.1	2.3
CASP-12 TOTAL	11.9	26.68	26.00	5.80	0.31	12	48	0.4	0.2

R: reversed item; SD: standard deviation.

**Table 3:** Convergent and internal validity of CASP-12.

	CASP-12				
	Control	Autonomy	Pleasure	Self-Realization	TOTAL
**Item-total corrected correlation**	0.47–0.56	0.13–0.26	0.31–0.43	0.48–0.61	0.25–0.59
**Internal validity**					
CASP-12 Autonomy	0.43 *	--	--	--	--
CASP-12 Pleasure	0.23 *	0.22 *	--	--	--
CASP-12 Self-realization	0.44 *	0.36 *	0.46 *	--	--
**Convergent validity**					
Socioeconomic status	0.12 *	0.22 *	0.09 *	0.14 *	0.19 *
Life satisfaction	0.35 *	0.38 *	0.36 *	0.46 *	0.52 *
GALI	−0.39 *	−0.20 *	−0.23 *	−0.34 *	−0.41 *
Self-perceived health	−0.41 *	−0.28 *	−0.21 *	−0.45 *	−0.47 *
Number of chronic diseases	−0.32 *	−0.22 *	−0.17 *	−0.32 *	−0.36 *
Euro-D	−0.49 *	−0.38 *	−0.30 *	−0.46 *	−0.57 *

Spearman’s rank correlation coefficients. * *p* < 0.01. GALI: Global Activity Limitation Indicator.

**Table 4:** Discriminative validity of CASP-12.

CASP-12					
	Control	Autonomy	Pleasure	Self-Realization	Total
By sex					
Men	8.33 (2.12)	8.18 (1.81)	9.54 (1.70)	8.16 (2.01)	34.23 (5.46)
Women	7.75 (2.29)	7.67 (1.86)	9.30 (1.83)	7.85 (2.08)	32.59 (5.97)
p ^a^	<0.001	<0.001	0.020	0.005	<0.001
By age					
50–64 years old	8.40 (2.16)	7.72 (7.97)	9.56 (1.72)	8.38 (1.93)	34.11 (5.41)
65–74 years old	8.04 (2.15)	7.97 (1.90) *	9.38 (1.74) *	7.93 (2.02)	33.31 (5.76)
>75 years old	7.18 (2.32)	8.09 (1.96)	9.17 (1.92)	7.36 (2.17)	31.80 (6.32)
p ^b^	<0.001	0.015	0.010	<0.001	<0.001
By GALI					
Not limited	9.02 (1.85)	8.36 (1.78)	9.90 (1.55)	8.81 (1.78)	36.10 (4.71)
Limited	7.28 (2.21)	7.57 (1.84)	9.05 (1.84)	7.40 (2.03)	31.33 (5.69)
p ^a^	<0.001	<0.001	<0.001	<0.001	<0.001
By self-perceived health					
Excellent to good	8.97 (1.85)	8.47 (1.72)	9.83 (1.59)	9.05 (1.73)	36.34 (4.76)
Fair	8.08 (2.02)	7.88 (1.80)	9.42 (1.72)	7.92 (1.82)	33.34 (5.05)
Poor	6.18 (2.26)	6.95 (1.82)	8.65 (1.97)	6.36 (2.00)	28.15 (5.58)
p ^b^	<0.001	<0.001	<0.001	<0.001	<0.001
By number of chronic diseases					
0–1 chronic diseases	8.68 (2.07)	8.27 (1.81)	9.63 (1.70)	8.59 (1.88)	35.21 (5.26)
2 or more chronic diseases	7.63 (2.24)	7.69 (1.85)	9.28 (1.80)	7.66 (20.7)	32.27 (5.83)
p ^a^	<0.001	<0.001	0.001	<0.001	<0.001

Mean (standard deviation). ^a^ Mann–Whitney test; ^b^ Kruskal–Wallis test with Bonferroni correction (* significant differences with the other groups). GALI: Global Activity Limitation Indicator.

**Table 5:** Internal consistency of CASP-12.

	Cronbach’s Alpha	Inter-Item Correlation	Item Homogeneity Index
CASP-12 Control	0.69	0.36–0.48	0.43
CASP-12 Autonomy	0.37	0.10–0.26	0.16
CASP-12 Pleasure	0.54	0.18–0.34	0.28
CASP-12 Self-realization	0.73	0.40–0.58	0.48
CASP-12 total ^a^	0.78	0.05–0.58	0.24

^a^ For all items of the scale.

**Table 6:** Fit of the CASP-12 domains to the Rasch model.

		Standard Values	Control	Autonomy	Pleasure	Self-Realization
Item fit residual	Mean	0	0.692	0.500	0.413	0.147
	SD	1	0.027	1.569	0.597	1.407
Person residual	Mean	0	0.236	0.189	1.096	0.048
	SD	1	1.429	0.903	1.123	1.873
Chi-square	Value		14.806	18.512	27.789	16.113
	Probability	>0.05/number of items	0.252	0.101	0.0267	0.186
Person Separation Index		>0.70	0.617	0.312	0.372	0.789

SD: standard deviation.

## Supplementary Materials

The following are available online at https://www.mdpi.com/1660-4601/17/18/6610/s1, **Table S1**: Summary of confirmatory factor analysis with 1, 3 and 4 factors.
