# Supplementary material for: Psychometric Properties of the CASP-12 Scale in Portugal: An Analysis Using SHARE Data
Source: Int J Environ Res Public Health. 2020 Sep 11;17(18):6610. doi: 10.3390/ijerph17186610 (PMC7558648; doi:10.3390/ijerph17186610)
Supplement: Supplementary file 1 [file ijerph-17-06610-s001.pdf]

Supplementary material

Table S1. Summary of confirmatory factor analysis with 1, 3 and 4 factors.

Loadings	1-factor model	4 factors		3 factors	
**1. Age prevents from doing things (ac014_)**	**0.53**	Control	0.63	Control + autonomy	0.62
2. Out of control (ac015_)	0.53		0.70		0.67
3. Feel left out of things (ac016_)	0.54		0.65		0.64
4. Do the things you want to do (ac017_r)	0.54	Autonomy	0.51		0.50
5. Family responsibilities prevent (ac018_)	0.24		0.25		0.30
6. Shortage of money stops (ac019_)	0.29		0.31		0.34
7. Look forward to each day (ac020_r)	0.33	Pleasure	0.43	Pleasure	0.43
8. Life has meaning (ac021_r)	0.58		0.75		0.76
9. Look back on life with happiness (ac022_r)	0.32		0.43		0.43
10. Feel full of energy (ac023_r)	0.70	Self-realization	0.68	Self-realization	0.67
11. Full of opportunities (ac024_r)	0.63		0.71		0.71
12. Future looks good (ac025_r)	0.63		0.70		0.7
**Fit indexes**					
χ2 (df)	850.77 (54)		444.59 (48)		497.42 (51)
RMSEA	0.100		0.075		0.077
CFI	0.791		0.896		0.883
RMSR	0.064		0.046		0.050
R^2	0.827		0.954		0.952

Df: degrees of freedom; RMSEA: root mean square error of approximation; CIF: comparative fit index; RMSR: standardized mean-square residual.
